# Supplementary material for: Pivotal relationship between heavy metal, PM2.5 exposures and tuberculosis in Bangladeshi children: protocol paper of a case–control study
Source: BMJ Open. 2024 Feb 2;14(2):e075010. doi: 10.1136/bmjopen-2023-075010 (PMC10840056; doi:10.1136/bmjopen-2023-075010)
Supplement: Author's manuscript [file bmjopen-2023-075010.draft_revisions.pdf]

BMJ Open

BMJ Open is committed to open peer review. As part of this commitment we make the peer review history of every article we publish publicly available.

When an article is published we post the peer reviewers' comments and the authors' responses online. We also post the versions of the paper that were used during peer review. These are the versions that the peer review comments apply to.

The versions of the paper that follow are the versions that were submitted during the peer review process. They are not the versions of record or the final published versions. They should not be cited or distributed as the published version of this manuscript.

BMJ Open is an open access journal and the full, final, typeset and author-corrected version of record of the manuscript is available on our site with no access controls, subscription charges or pay-per-view fees (http://bmjopen.bmj.com).

If you have any questions on BMJ Open's open peer review process please email info.bmjopen@bmj.com

BMJ Open

The pivotal relationship between heavy metal exposures and tuberculosis in Bangladeshi children: Protocol paper of a case-control study

Journal:	*BMJ Open*
Manuscript ID	bmjopen-2023-075010
Article Type:	Protocol
Date Submitted by the Author:	24-Apr-2023

Complete List of Authors:	Sarah, Rehnuma; International Centre for Diarrhoeal Disease Research Bangladesh, Environmental Interventions Unit; Uppsala Universitet, Dept. of Women’s and Children’s Health<br>Hanson, Molly; Uppsala Universitet, Department of Women's and Children's Health<br>Shariful Islam, Md ; International Centre for Diarrhoeal Disease Research Bangladesh, Environmental Interventions Unit; The University of Queensland, The department of Public Health<br>Akter, Nazrin; International Centre for Diarrhoeal Disease Research Bangladesh, Environmental Interventions Unit<br>Moniruzzaman, Mohammad ; Bangladesh Council of Scientific and Industrial Research<br>Alam, Md. Jahangir; Dhaka Shishu Hospital<br>Kamruzzaman, Md.; Dhaka Shishu Hospital<br>Rahman, Mahbubur ; International Centre for Diarrhoeal Disease Research, Environmental Interventions Unit<br>Chisti, Md.; International Centre for Diarrhoeal Disease Research, Bangladesh (icddr,b), Nutrition and Clinical Services Division<br>Raqib, Rubhana; International Centre for Diarrhoeal Disease Research Bangladesh<br>Rahman, Syed; Uppsala University, International Maternal and Child Health, Department of Women's and Children's Health

Keywords:	Tuberculosis < INFECTIOUS DISEASES, EPIDEMIOLOGIC STUDIES, Community child health < PAEDIATRICS, TOXICOLOGY, Immunology < TROPICAL MEDICINE

SCHOLARONE™ Manuscripts

The pivotal relationship between heavy metal exposures and tuberculosis in Bangladeshi children: Protocol paper of a case-control study

Rehnuma Haque Sarah[1,4], Molly Hanson[4], Md. Shariful Islam[5], Nazrin Akter[1], Md. Moniruzzaman[3], Md. Jahangir Alam[2], Md. Kamruzzaman[2], Mahbubur Rahman[1], Mohammod Jobayer Chisti[1], Rubhana Raqib[1], Syed Moshfiqur Rahman[1,4]

1. icddr,b, 68 Shahed Tajudin Ahmed Sarani, Mohakhali, Dhaka, Bangladesh, 1212
2. Bangladesh Shishu Hospital & Institute, Sher-e-Bangla Nagar, Dhaka-1207, Bangladesh.
3. Bangladesh Council of Scientific and Industrial Research (BCSIR)
4. Department of Women's and Children's Health, Uppsala University, 752 36 Uppsala, Sweden
5. The School of Public Health, The University of Queensland, Brisbane, Qld, Australia

Corresponding author: Rehnuma Haque Sarah, Bangladesh, Dept. of Women's and Children's Health | Uppsala University, +8801755636851, rehnuma.haque@icddrb.org rehnuma.haque@uu.se

Keyword: Tuberculosis, air pollution, heavy metals, Children, LMICs

ABSTRACT

Introduction: Air pollution is a global issue that poses a significant threat to public health. Children, due to their developing physiology, are particularly susceptible to the inhalation of environmental pollutants. Exposure can trigger immune modulation and organ damage, increasing susceptibility to respiratory diseases. Therefore, we aim to examine the association between heavy metal and particulate matter exposure with tuberculosis in children.

Methods and Analysis: As a case-control study, we will include children diagnosed with pulmonary tuberculosis (n=60) and matched healthy controls (n=80) recruited from the same communities in Dhaka, Bangladesh. Exposure data for both cases and controls will be collected by a trained field team conducting home visits. They will administer an exposure questionnaire, measure child anthropometry, collect blood and household dust samples and install 48-hour air quality monitors. The blood samples will be analysed by Inductively Coupled Plasma Mass Spectrometry for serum heavy metal concentrations (Pb, Cd, As, Hg and Cr), as a representative marker of exposure, and the presence of inflammatory biomarkers. Descriptive and inferential statistics, including independent samples t-tests, ANOVA and generalized additive modelling, will be used to quantify heavy metal and particulate matter exposure status in tuberculosis cases compared to healthy controls, while accounting for potential confounders. Dust samples and air quality results will be analysed to understand household sources of heavy metal and particulate matter exposure. To test the study hypothesis, that there is a positive association between exposure and tuberculosis infection, we will also measure the accumulated effect of simultaneous exposures using Bayesian statistical modelling.
Ethics and Dissemination:

For peer review only - http://bmjopen.bmj.com/site/about/guidelines.xhtml

Article Summary

Strengths and Limitations of this study

- Few case-control studies on a target population of children in highly polluted location.
- Pilot study designed with a small sample size.
- Extensive data collection for a variety of exposures and to control for confounders.
- Unable to differentiate between other sources of exposure, including via food and drinking water.
- Skewed blood metal concentrations will be log-transformed or categorized, potentially resulting in the loss of specific details.

INTRODUCTION

Low- and middle-income countries (LMICs) face the highest burden of both environmental pollution and respiratory infections. The World Health Organization (WHO) estimated globally more than one million children and young adolescents under 15 years fall ill with tuberculosis (TB) every year, and a quarter of those die (World Health Organization 2022). An estimated 35,000 children with TB live in Bangladesh. However, determining the underlying causes and preventing TB in children are neglected as global health priorities and remain major challenges for Bangladesh (Kak et al. 2020). Similar to many LMICs, Bangladesh is also overburdened by environmental toxicants and pollutants, both naturally occurring and exacerbated by anthropogenic activities. There is growing evidence of high exposure to environmental pollutants in Bangladesh, through drinking water, soil, food, dust and ambient air (Islam et al. 2018).

Vehicle emissions, spraying pesticides and fertilizers, industrial processes, mining, cooking and combustion are widespread anthropogenic sources of heavy metal pollutants, including arsenic (As), cadmium (Cd), lead (Pb), mercury (Hg) and chromium (Cr) (Sarker et al. 2022; World Health Organization 2018). In Bangladesh, there are health concerns regarding the contamination of common food products and drinking water with heavy metals (Sarker et al. 2022). In addition, in 2020, AirVisual classified air quality in Bangladesh as 'unhealthy' (IQAir 2023). Small airborne particle mixtures of heavy metals and organic species, such as carbon compounds, constitute respirable particulate matter (PM) (Sarker et al. 2022; Tarannum et al. 2021). Living in Dhaka is a significant risk factor for exposure to air pollutants, as the capital city scores almost twenty times higher than the WHO recommended level of PM (Tarannum et al. 2021; IQAir 2023). Therefore, through overlapping pathways of ingestion, inhalation and dermal absorption, exposure to potentially harmful levels of PM and heavy metals in Bangladesh is inevitable.

Airborne particles of heavy metals and PM cross from the respiratory system into the blood circulation (Sarker et al. 2022; Tarannum et al. 2021). The mechanisms of toxicity are not fully understood but contribute to inflammation, tissue damage and immune modulation, as described in *figure 1* (Balali-Mood et al. 2021). Children are particularly susceptible to this environmental pollution toxicity due to their under-developed physiology and immature immune systems

For peer review only - http://bmjopen.bmj.com/site/about/guidelines.xhtml

(World Health Organization 2018; Landrigan et al. 2019). A growing body of research shows that children's lung growth and function is significantly worse in areas with higher air pollution (World Health Organization 2018; Landrigan et al. 2019). Simultaneous exposure to multiple environmental pollutants and the interaction between heavy metals may also have synergistic effects (Balali-Mood et al. 2021; Ackland et al. 2015). Therefore, exposure to PM and heavy metals may alter the immune system, increasing susceptibility to pathogens and respiratory diseases, such as *Mycobacterium tuberculosis* bacteria (Landrigan et al. 2019; Davis and Checkley, n.d.; Ackland et al. 2015).

This paper describes the design of a patient-oriented pilot study which seeks to uncover the interplay and potential causal pathway connecting environmental heavy metals to tuberculosis. The rationale for the proposed study builds on existing research, highlighting that exposure to PM is significantly associated with an increased incidence of pulmonary tuberculosis (PTB) in adults (Dimala and Kadia 2022). However, the current body of evidence is limited on the environmental risks for children in Bangladesh. Furthermore, significantly greater progress is required to achieve the UN Sustainable Development Goal, target 3.3, to end tuberculosis epidemics by 2030 (United Nations Statistical Division 2018). We hypothesise that there is a positive association between PM and heavy metal exposure and PTB infection in children. This study aims to bridge the current research gap, to understand the role of pollutants in TB pathogenesis, which is crucial to develop appropriate public health interventions and prevention activities.

We will achieve this with the following study objectives, to

(1) test the hypothesis that exposure to heavy metals (e.g., Pb, As, Cd, Hg and Cr) and PM is a risk factor for tuberculosis in children and
(2) quantify environmental sources of heavy metals, and PM exposure among children in urban areas in Dhaka city.

Conceptual framework

Based on existing literature and evidence, this aim reflects a potential causal pathway (*figure 1*) connecting inhalation of airborne pollutants with alterations in the immune system response. Toxic metals constitute ultrafine particulate matter ($PM_{2.5}$), considered the most harmful to the respiratory system, as the particles can deposit in the airways or cross the air-blood barrier in the lungs (Balali-Mood et al. 2021; Ackland et al. 2015). In the blood stream, Pb, As, Cd, Hg and Cr accumulate and have been shown to induce toxicity by generating reactive-oxygen species (ROS). ROS suppress the body's antioxidant defence, induce oxidative stress and alter the activation of specific proteins, lipids and enzymes involved in cell defence and the immune response (Balali-Mood et al. 2021). Toxic metals disrupt immune homeostasis, triggering excessive production of immunoglobulins (IgE and IgA) and pro-inflammatory cytokines (CD8+) and NK cells while decreasing memory B and T lymphocytes (Ackland et al. 2015). These overlapping pathways result in persisting inflammation, immune modulation and organ toxicity.

TB pathogenesis can be attributed to increased susceptibility to infectious diseases and impairment in lung function (Balali-Mood et al. 2021; Ackland et al. 2015). Due to their under-

For peer review only - http://bmjopen.bmj.com/site/about/guidelines.xhtml

developed physiology, children's immature lungs are uniquely susceptible to respiratory tract infections (World Health Organization 2018; Ackland et al. 2015). Children are crawling, playing and breathing in the air closest to the ground, where high levels of pollutants are concentrated (World Health Organization 2018). In contrast to adults, children under five have a faster breathing rate and a relatively immature immune system, making them less efficient in responding to pollutants and pathogens (Landrigan et al. 2019).

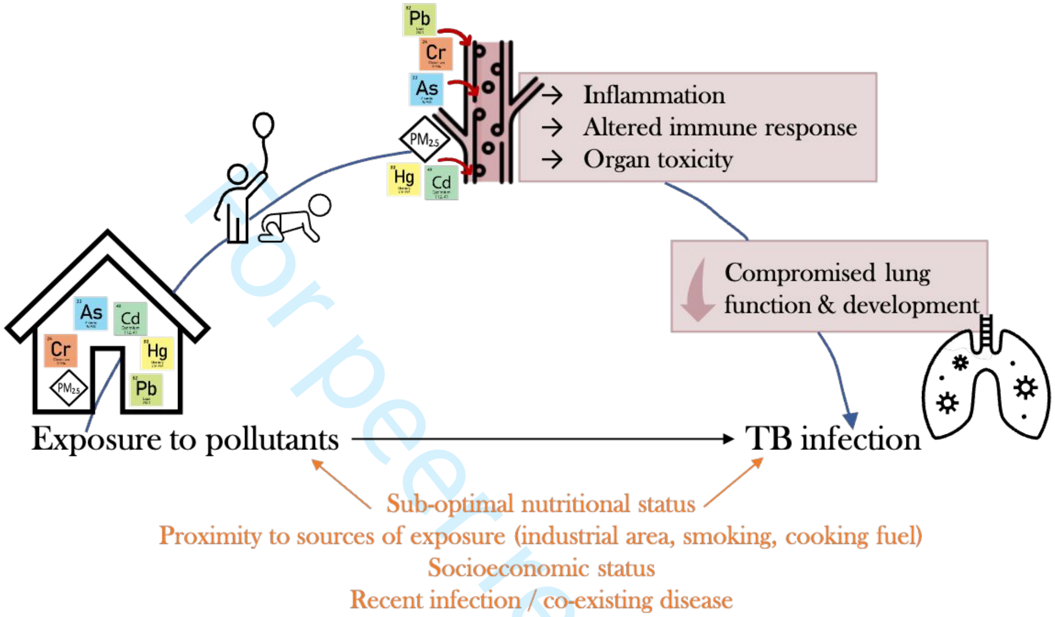

Figure 1: Proposed causal pathway, adapted from existing literature (World Health Organization 2018; Balali-Mood et al. 2021; Landrigan et al. 2019; Davis and Checkley, n.d.; Ackland et al. 2015)

METHODS

Study design and setting

We will conduct a case-control study to compare exposure to heavy metals among children diagnosed with TB (case) and matched healthy controls in different urban areas of Dhaka City, Bangladesh.

The population of Dhaka has multiplied over 4 times since 1980, attaining one of the highest population densities in the world, with a remarkable 24 million inhabitants. With persisting urbanisation attracting people to the city, this number is expected to continue growing. As a consequence, rapid urbanisation has aggravated the concentration of pollutants in air, soil and water (Bird et al. 2018). Bangladesh, and especially the capital city of Dhaka, has recorded some of the worst air pollution in the world (IQAir 2023). Existing studies suggest wide-ranging health consequences from exposure to heavy metals and PM through ingestion, inhalation and dermal absorption (Islam et al. 2018; Sarker et al. 2022; Tarannum et al. 2021). Bangladesh was selected as the study site as it shares one of the highest burdens of tuberculosis infections in the world (Kak et al. 2020). The study is hosted by the Environmental Interventions Unit (EIU) of the Infectious Diseases Division, icddr,b.

For peer review only

We will measure whole blood for heavy metal concentrations to assess exposure and conduct immunological assays to evaluate immune status of the patient. We will also conduct household air and dust sampling to identify some sources of heavy metal exposure among children. Household geo-mapping, an exposure questionnaire and socio-economic surveys from the parents will be used to further explore the sources of metal exposure among the children. Written consent will be obtained from guardians/parents and assent from older children (10-14 years) before the data and specimen collection.

Study participants

We will recruit children between the ages of 1 and 14 living in Dhaka city who have been diagnosed with pulmonary tuberculosis (PTB) by a physician. TB cases will be identified based on the WHO definition, which refers to any bacteriologically confirmed *Mycobacterium tuberculosis* infection that affects the lung parenchyma or tracheobronchial tree without radiographic abnormalities in the lungs (World Health Organization 2022).

At the beginning of the project, we will visit the TB unit of Dhaka Shishu Hospital, which is specifically designated for pediatric TB patient's ongoing diagnosis and treatment. To identify eligible participants, we will obtain a registry of TB-diagnosed children, providing the names, ages, addresses and contact details of children diagnosed with PTB. We will then contact the parents or caregivers to request for their child's participation in our study.

Following our inclusion and exclusion criteria, we will enrol 50 children diagnosed with pulmonary TB. In addition, we will then select 50 healthy controls between the ages of 1 and 14 years old who live in the same communities as the cases. According to Rothman et al., it is important to match cases with the controls from similar geographic locations as a partial adjustment of environmental and socio-economic factors (Rothman, Greenland, and Lash 2008). The process of selecting controls will be carried out carefully to minimize bias and ensure the validity of our study results. We will identify potential controls by conducting a door-to-door survey within a half-kilometer radius of each case child's residence. We will select the first subsequent child of the same age who participates in shared activities, such as schooling, playing, learning, or mosque visits. To ensure the control children are healthy, the exclusion criteria for controls will be: (i) hospitalisation in the past 60 days, and/or discharged from hospital in the past 30 days; (ii) known chronic disease; (iii) very sick appearance requiring medical attention; (iv) recent cough with blood or painful ear infections; or (v) recovery from diagnosed TB.

To account for potential dropouts, we aim to enrol a total of 60 children in the case group and 80 children in the control group.

Sample size calculation

To determine our required sample size, we have considered the sample size for similar case-control studies with continuous exposures, as described by Lubin et al., (Lubin, Gail, and Ershow 1988). The formula used to calculate sample size was:

For peer review only - http://bmjopen.bmj.com/site/about/guidelines.xhtml

$$n = \left(\frac{k+1}{k}\right)\frac{\overline{p}(1-\overline{p})(z_\beta + z_{\alpha/2})^2}{(p_1 - p_2)^2}$$

Where,
k = ratio of control to case,
p_1 = proportion of cases exposed,
p_2 = proportion of controls exposed,
ß = power,
α = level of significance,
$\overline{p} = \frac{(p_1 + p_2)}{2}$

We estimate that a sample size of 50 children, in both the case and control groups, would achieve 80% power to detect a mean difference in blood concentrations and odds ratio of 5 at a significance level of 0.05. Recruiting 60 children in the case group and 80 children would allow for a drop out of 10 and 20 children in each group respectively.

Exposure questionnaire and anthropometry
Over a period of six to eight months, a trained field team will conduct home visits and administer a questionnaire to the mother or primary caregiver on household exposures. The questionnaire will cover a range of topics including socioeconomic status (SES), demographics, sources of environmental and household pollution (such as smoking, type of cooking fuel, proximity to battery/textile/waste incineration areas), and other behaviors that may impact pollutant exposure (such as engagement in industrial manufacturing or agricultural practices). Additionally, we will inquire about the children's medical history, including any recent infections or co-existing conditions.

An experienced medical technologist will also gather anthropometric data to assess the child's overall nutritional status. The child's height and weight will be measured using a standardized wooden height scale and digital weight scale (Beurer ps240). These will be validated for accuracy and precision at icddr,b before going to the field.

Clear instructions will be provided to the child prior to weighing, emphasizing the importance of removing any items that could affect the measurement, such as shoes or heavy jackets. The child will be positioned with their weight evenly distributed on both feet, and they will be asked to stand still on the weighing machine with their arms at their sides. The weight measurement will be taken in kilograms and repeated. If the two measurements are noticeably different, we will repeat the process and calculate the average of three measurements to ensure accuracy. Similarly, when measuring height, the child will remove any items that may affect the measurement, such as shoes, socks, headgear, or hair accessories. The child will be informed to stand upright and look straight ahead. Measurements will be taken in centimeters. For infants under 2 years of age, or who cannot stand, we will adjust the anthropometric measurements by laying them on a flat surface for height and in a weighing tray for weight.

Biological sample collection

For peer review only - http://bmjopen.bmj.com/site/about/guidelines.xhtml

During the household visits, the field team will also collect blood samples, household dust and indoor air samples, from both case and control groups. These samples will be analyzed to compare biomarkers and environmental exposures between the two groups. By characterizing the sources and risk of pollutant exposure, we can propose intervention strategies to reduce the burden of paediatric TB in low- and middle-income countries.

To collect the blood sample, the area around the cubital vein will first be sterilized. We will use a S-Monovette® Metal analysis Lithium-Heparin 7.5 ml tube and Safety-Multifly® needle to draw 5ml of blood from the child's vein. After collecting the blood, it will be placed in a sterile tube and transported in a cooler box to the Immunobiology, Nutrition and Toxicology Laboratory at icddr,b for analysis. Upon arrival at the laboratory, each sample will be carefully labeled with a unique barcode and anonymous relevant information, including the date of collection and household ID. The sample tube will then be placed in the collected blood in rotator for five minutes, after which 1 ml of whole blood will be separated and placed in a cryovial. The remaining blood will be centrifuged into the separate serum, plasma and red blood cell components, with each stored in a cryovial. Blood samples will be stored at -80◦C, complying with the icddr,b data storage policy, for relevant analysis depending on the future availability of funds.

For heavy metal concentrations in serum, the primary exposures of interest are lead (Pb) and cadmium (Cd), while secondary exposures include arsenic (As), mercury (Hg) and chromium (Cr). Aliquots of blood will be analysed for these metals by Inductively Coupled Plasma Mass Spectrometry (ICP-MS). The serum will be preserved for future Immunoassays for determining the presence of inflammatory and nutritional markers. We will conduct all laboratory analyses in duplicate and include field blanks to ensure sterile sample collection and processing.

Environmental sample collection

We will install portable air quality monitors (PurpleAir) in participants' homes to measure real-time $PM_{2.5}$ levels for at least 48 hours. They will be placed in the child's bedroom or other places where they spend most of their time at home. Data on $PM_{2.5}$, humidity, and temperature will be sent directly to our smart devices.

During the household visits, the team will collect samples of household dust from the children's homes. These will be analysed at the Environmental Interventions Unit, icddr,b using x-ray fluorescence spectrometry (XRF). A subset of dust samples will be sent to the Bangladesh Council of Scientific and Industrial Research (BCSIR) laboratory for quality assurance, where repeat measures will be performed by ICP-MS and data will be compared with XRF data. The results will be used to fulfil the second research objective, to identify and quantify environmental sources of heavy metals and PM.

Data management and analysis plan

A data management standard operating protocol will be developed considering the range and variation in data types. Field researcher officers will consult with the research assistants on a daily basis to check data consistency. The household questionnaire data will be digitally programmed through tablet computers, downloaded from the devices each week and reviewed by an Environmental Interventions Unit (EIU) statistician. The statistician will update the server and

For peer review only - http://bmjopen.bmj.com/site/about/guidelines.xhtml

produce datasets for the investigators in R programme language. Once the data are available to the investigators, they will run previously developed Stata do files to assess inconsistencies and send queries to the field team. All metadata will be stored on the EIU's Data Repository System and a backup will be taken.

As evidenced in similar studies, the distributions of Pb, As, Cd, Hg and Cr in the blood samples are expected to be skewed. In order to fulfil the assumptions of the selected parametric tests, this data will be transformed, by either natural log transformation or categorisation of the metal concentrations.

Descriptive analysis, including frequency tests, will be stratified by child's age, sex and TB condition. Chi-squared, independent samples t-tests or ANOVA will be performed to compare the blood heavy metal concentrations among TB cases and healthy controls. In addition, we will compare the sociodemographic information, based on the parental exposure questionnaire survey. We will run a generalized additive model (GAM) to estimate the biomarkers that are associated with heavy metal and PM exposures among TB children. In addition, if we identify two or more elevated metals in the blood and/or environmental samples, we will measure their accumulated effect using Bayesian statistical modelling (Shah-Kulkarni et al. 2020).

Ethical considerations

The study design and protocols for data collection will be submitted for review by the Institutional Review Board (IRB) of icddr,b consisting of the Research Review Committee (RRC) and the Ethical Review Committee (ERC). The study will be initiated after it has been approved by the IRB. The investigators who will be managing and analyzing the human data will have up-to-date ethics certification.

There are minor risks to the participants during sample collection. The risks and advantages for participants will be provided in written information forms to ensure the participants are fully informed about the study. The purpose and methods of the study will also be clearly described. This information will be written in English and translated into Bangla in a format that is easy to understand even with little or no educational background. If required, the form will also be read out to the participant.

As this study recruits children between one and 15 years of age, the mothers or caregivers will be required to give consent on behalf of their children participating in the study. Before taking their consent, the researchers will repeat the study information and participants will have time to ask any questions. The participants will also be informed that they have the freedom to withdraw from the study at any time and have no adverse implications. Those who agree to participate will be required to give written informed consent, by signing or making an imprint of their left thumb and verified by an independent witness. Participants will also keep a copy of the information and consent forms so they have open access and can contact the research review committee if necessary.

Field data and the sample test results will be uploaded to the secured server at icddr,b. Only investigators will have access to the dataset. Prior to uploading the data, the field supervisor will check the datasets for consistency and identify any missing information. If any inconsistencies

For peer review only - http://bmjopen.bmj.com/site/about/guidelines.xhtml

are identified, queries will be sent to the field and resolution of the discrepancies will be made. Throughout the data management, confidentiality of information will be strictly maintained, and access to the data will be restricted to the research team members only. At the time of analyzing information and publishing the results of the study, the name or identity of the study participants will not be used.

Expected results

The hypothesis-driven research we propose is intended to address the gap in developing childhood TB infection and the association with environmental pollutant exposure, with a focus on air pollution and heavy metal exposure. This research will provide evidence for follow-up studies to improve understanding of the causal pathway connecting these pollutants to TB infection and inform evidence-based guidance to eliminate TB. Our long-term goal is to evaluate the chemical risk factors for respiratory infections and to improve children's health.

Declaration of competing interest

The authors declare that they have no known competing financial interests or personal relationships that could have appeared to influence the work reported in this paper.

Acknowledgements

We express our gratitude to the governing body for granting us permission to carry out patient enrollment at Dhaka Shishu Hospital and access to laboratory facilities at Bangladesh Council of Scientific and Industrial Research (BCSIR) for our research endeavors.

Funding

This research is funded by Thrasher Research Fund- Early Career Award number 01477.

REFERENCES

Ackland, M. Leigh, Julia Bornhorst, George V. Dedoussis, Rodney R. Dietert, Jerome O. Nriagu, Jozef M. Pacyna, and John M. Pettifor. 2015. 'Metals in the Environment as Risk Factors for Infectious Diseases: Gaps and Opportunities'. In *Trace Metals and Infectious Diseases*, edited by Jerome O. Nriagu and Eric P. Skaar. Cambridge (MA): MIT Press. http://www.ncbi.nlm.nih.gov/books/NBK569691/.

Balali-Mood, Mahdi, Kobra Naseri, Zoya Tahergorabi, Mohammad Reza Khazdair, and Mahmood Sadeghi. 2021. 'Toxic Mechanisms of Five Heavy Metals: Mercury, Lead, Chromium, Cadmium, and Arsenic'. *Frontiers in Pharmacology* 12. https://www.frontiersin.org/articles/10.3389/fphar.2021.643972.

Bird, Julia, Yue Li, Hossain Zillur Rahman, Martin Rama, and Anthony J. Venables. 2018. *Toward Great Dhaka: A New Urban Development Paradigm Eastward*. Washington, DC: World Bank. https://doi.org/10.1596/978-1-4648-1238-5.

Davis, J. Lucian, and William Checkley. n.d. 'Characterization of Air Pollution Exposures as Risk Factors for Tuberculosis Infection'. *American Journal of Respiratory and Critical Care Medicine* 204 (10): 1130–31. https://doi.org/10.1164/rccm.202107-1795ED.

For peer review only - http://bmjopen.bmj.com/site/about/guidelines.xhtml

Dimala, Christian Akem, and Benjamin Momo Kadia. 2022. 'A Systematic Review and Meta-Analysis on the Association between Ambient Air Pollution and Pulmonary Tuberculosis'. *Scientific Reports* 12 (1): 11282. https://doi.org/10.1038/s41598-022-15443-9.

IQAir. 2023. 'Dhaka Air Quality Index (AQI)'. Air Quality Index (AQI) and PM2.5 Air Pollution in Dhaka. 1 February 2023. https://www.iqair.com/bangladesh/dhaka.

Islam, M. Mominul, Md. Rezaul Karim, Xin Zheng, and Xiaofang Li. 2018. 'Heavy Metal and Metalloid Pollution of Soil, Water and Foods in Bangladesh: A Critical Review'. *International Journal of Environmental Research and Public Health* 15 (12): 2825. https://doi.org/10.3390/ijerph15122825.

Kak, Neeraj, Krishnapada Chakraborty, Swati Sadaphal, Hala Jassim AlMossawi, Marianne Calnan, and Begum Vikarunnessa. 2020. 'Strategic Priorities for TB Control in Bangladesh, Indonesia, and the Philippines – Comparative Analysis of National TB Prevalence Surveys'. *BMC Public Health* 20 (April): 560. https://doi.org/10.1186/s12889-020-08675-9.

Landrigan, Philip J., Richard Fuller, Samantha Fisher, William A. Suk, Peter Sly, Thomas C. Chiles, and Stephan Bose-O'Reilly. 2019. 'Pollution and Children's Health'. *Science of The Total Environment* 650 (February): 2389–94. https://doi.org/10.1016/j.scitotenv.2018.09.375.

Lubin, J. H., M. H. Gail, and A. G. Ershow. 1988. 'Sample Size and Power for Case-Control Studies When Exposures Are Continuous'. *Statistics in Medicine* 7 (3): 363–76. https://doi.org/10.1002/sim.4780070302.

Rothman, Kenneth J., Sander Greenland, and Timothy L. Lash. 2008. *Modern Epidemiology*. 3rd ed. Philadelphia: Lippincott Williams & Wilkins.

Sarker, Aniruddha, Jang-Eok Kim, Abu Reza Md. Towfiqul Islam, Muhammad Bilal, Md. Refat Jahan Rakib, Rakhi Nandi, Mohammed M. Rahman, and Tofazzal Islam. 2022. 'Heavy Metals Contamination and Associated Health Risks in Food Webs—a Review Focuses on Food Safety and Environmental Sustainability in Bangladesh'. *Environmental Science and Pollution Research* 29 (3): 3230–45. https://doi.org/10.1007/s11356-021-17153-7.

Shah-Kulkarni, Surabhi, Seulbi Lee, Kyoung Sook Jeong, Yun-Chul Hong, Hyesook Park, Mina Ha, Yangho Kim, and Eun-Hee Ha. 2020. 'Prenatal Exposure to Mixtures of Heavy Metals and Neurodevelopment in Infants at 6 Months'. *Environmental Research* 182 (March): 109122. https://doi.org/10.1016/j.envres.2020.109122.

Tarannum, Maliyat, Abdus Salam, Farah Jeba, Md Riad Sarkar Pavel, and Shahid Uz Zaman. 2021. 'Health Risk Assessment of High-Level Particulate Matter Exposure in Different Environments in Mega City Dhaka, Bangladesh'. *The Bangladesh Journal of Scientific Research*, July, 78–87.

United Nations Statistical Division. 2018. '3.3.2 Tuberculosis Incidence'. Global SDG Indicator Platform. 31 October 2018. https://sdg.tracking-progress.org/indicator/3-3-2-tuberculosis-incidence/.

World Health Organization. 2018. 'Air Pollution and Child Health: Prescribing Clean Air'. Summary. Geneva.

———. 2022. 'WHO Consolidated Guidelines on Tuberculosis: Module 5: Management of Tuberculosis in Children and Adolescents'. World Health Organization. https://www.who.int/publications-detail-redirect/9789240046764.

For peer review only - http://bmjopen.bmj.com/site/about/guidelines.xhtml

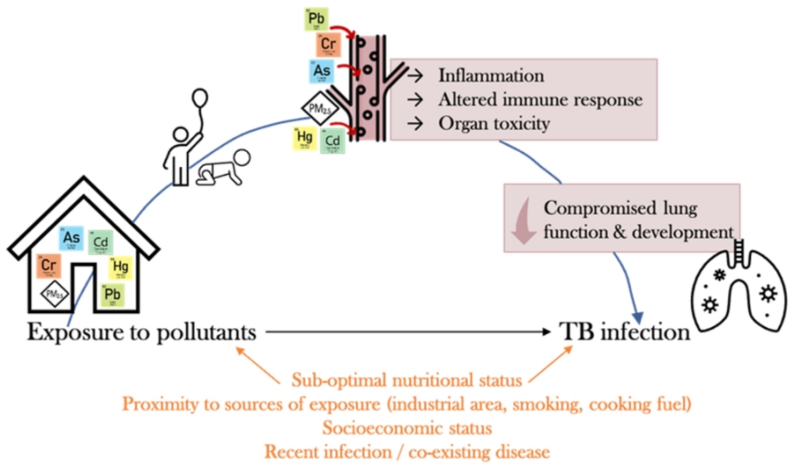

855x481mm (38 x 38 DPI)

Ethical Review Committee
Approval Letter

26 May 2022

To: Dr Rehnuma Haque
Principal Investigator of research protocol # PR-22030
Infectious Diseases Division (IDD)

From: Professor Ahmed Abu Saleh
Chairperson
Ethical Review Committee (ERC)

Sub: **Approval of research protocol # PR-22030**

Approval Date: 26 May 2022
Expiration Date: 25 May 2023
Review Type: Full Committee Review
Risk Level: No more than minimal
Project type: New Project

Thank you for your memo dated 22 May 2022 attaching the modified version of your research protocol # PR-22030, titled "Exploring the link of toxic metals exposure with tuberculosis in children"; version No. 2.0; version date 18 April 2022; addressing the issues raised by the ERC in its 40th Virtual ERC meeting held on 28 April 2022. I am pleased to inform you that your protocol is approved. You will be required to observe the following terms and conditions in implementing the research protocol:

1. The research protocol is approved for 12-month period from the date of approval of the protocol by the Ethical Review Committee. The Federal regulations require review of an approved study not less than once per 12-month period. To comply with federal regulations, a continuing review application must be submitted to the IRB Secretariat for this study to continue beyond 25 May 2023.

   All necessary materials for continuing review must be reviewed with sufficient time for review and issuing continued approval before the expiration date. Failure to initiate a continuing review application in a timely fashion may result in discontinuation of study activities until approval can be renewed. Performing study activities, including data analysis, beyond the expiration date results in noncompliance of federal regulations.

2. The ERC approval shall automatically be revoked after one year if the protocol is not started. After one year, you shall have to seek approval for revalidation of the protocol by the ERC before starting.

3. You should notify the IRB Secretariat of the start date of the protocol for updating in the integrated Navision system. The protocol start date will not be

Ethical Review Committee, IRB Secretariat, Research Administration, CMS
International Centre for Diarrhoeal Disease Research, Bangladesh
68, Shaheed Tajuddin Ahmed Sarani, Mohakhali, Dhaka 1212, Bangladesh
Mail: icddr,b, GPO Box 128, Dhaka 1000, Bangladesh
Phone: 880-2-9827084, Web: http://www.icddrb.org

For peer review only - http://bmjopen.bmj.com/site/about/guidelines.xhtml

updated in the Navision system until receiving information from you. Therefore, you will not be able to operate budget code and continue spending funds under the research protocol.

4. As Principal Investigator, the ultimate responsibility for scientific and ethical conduct including the protection of the rights and welfare of study participants vest upon you. You shall also be responsible for ensuring competence, integrity and ethical conduct of other investigators and staff directly involved in this research protocol.

5. You shall conduct the study in accordance with the ERC-approved protocol and shall fully comply with any subsequent determinations by the ERC.

6. You shall obtain prior approval from the Research Review Committee and the ERC for any modification in the approved research protocol and/or approved consent form(s), except in case of emergency to safeguard/ eliminate apparent immediate hazards to study participants. Such changes must immediately be reported to the ERC Chairman.

7. You shall recruit/enrol participants for this study strictly adhering to the criteria mentioned in the research protocol.

8. You shall obtain legally effective informed consent (i.e. consent should be free from coercion or undue influence) from the selected study participants or their legally responsible representative, as approved in the protocol, using the approved consent form prior to their enrolment in this study. Before obtaining consent, all prospective study participants must be adequately informed about the purpose(s) of the study, its methods and procedures, and also what would be done if they agree and also if they do not agree to participate in the study.

9. They must be informed that their participation in the study is voluntary and that they can withdraw their participation any time without any prejudice. Signed consent forms should be preserved for a period of at least five years following official termination of the study.

10. You shall promptly report the occurrence of any Serious Adverse Event or unanticipated problems of potential risk to study participants or others to the ERC in writing within 24 hours of such occurrences.

11. Any significant new findings, developing during the course of this study that might affect the risks and benefits and thus influence either participation in the study or continuation of participation should be reported in writing to the participants and the ERC.

12. You shall report progress of research to the ERC for continuing review of the implementation of the research protocol as stipulated in the ERC Guidelines. Relevant excerpt of ERC Guidelines and '*Annual/Completion* Report for Research Protocol involving Human Subjects' are attached for your information and guidance.

13. Data and/or samples should be collected and interviews should be conducted, as specified in the ERC-approved protocol, and confidentiality must be

For peer review only - http://bmjopen.bmj.com/site/about/guidelines.xhtml

maintained. Data/samples must be protected by reasonable security, safeguarding against risks such as their loss or unauthorized access, destructions, used by others, and modification or disclosure of data. Data/samples should not be disclosed, made available to or use for purposes other than those specified in the protocol, and shall be preserved for a period, as specified under Centre's policies/practices.

14. You shall promptly and fully comply with the decision of the ERC to suspend or withdraw its approval for the research protocol.

15. The ERC should be immediately notified if the protocol is discontinued before the expected date of completion.

Approved documents:

a. Protocol version No 2.0; dated 18 April 2022
b. English and Bangla Consent form for caregiver interviews, survey, sample collection, and child blood draw; Version No. 0.01; dated 30 December 2021
c. English and Bangla Assent form for < 15 children, survey, sample collection, and child blood draw (English); Version No. 0.01; dated 30 December 2021
d. Survey questionnaire;
e. Tuberculosis guidelines

The IRB of icddr,b shall take into account the regulations of the Bangladesh Medical Research Council (BMRC), WHO, international guidelines for biomedical research as laid down by the Council of International Organization of Medical Sciences (CIOMS), the Declaration of Helsinki in relation to biomedical research involving human participants, ICH Guidelines on Good Clinical Practice (GCP), National Institutes of Health (NIH), National Institute of Allergy and Infectious Diseases (NIAID), and Division of Microbiology and Infectious Diseases (DMID). If there is any new declaration involving human participants, contents of such declaration should be appropriately adhered to and the applicable laws and policies of the local government.

I wish you success in running the above-mentioned study.

Cc: Senior Director, IDD
Senior Manager, Budget & Planning, Finance

For peer review only - http://bmjopen.bmj.com/site/about/guidelines.xhtml

Cover Letter

Date: April 23, 2023
Editor-in-Chief, BMJ Open
Re: Submission of a protocol paper titled " The pivotal relationship between heavy metal exposures and tuberculosis in Bangladeshi children".

Dear Editor,

We are pleased to submit a protocol paper titled "The Crucial Link between Heavy Metal Exposure and Tuberculosis in Children in Bangladesh." Our study is the first of its kind to examine the impact of air PM2.5 and PM-bound heavy metal concentrations on children with tuberculosis in Bangladesh. Our aim is to investigate the ways in which environmental pollutants contribute to the development, propagation, and exacerbation of infectious diseases. As a result of rapid urbanization, developing countries are confronted with the dual challenges of infectious diseases and pollution.

We are confident that our research will be of great interest to BMJ Open readers, particularly those engaged in uncovering the causal connections between environmental pollution, climate change, and communicable diseases, especially in low-income urban areas. We confirm that the material presented in this manuscript is original and has not been previously published, nor is it being considered for publication elsewhere.

Thank you for considering our protocol paper for publication.

Sincerely,

Rehnuma Haque Sarah
Assistant Scientist, Infectious Diseases Division
International Centre for Diarrhoeal Disease Research, Bangladesh (icddr,b)
Affiliated: Adjunct Scientist, Dept of Women and Children health
Uppsala University, Sweden
Email: rehnuma.haque@uu.se

For peer review only

BMJ Open

The pivotal relationship between heavy metal exposures and tuberculosis in Bangladeshi children: Protocol paper of a case-control study

Journal:	*BMJ Open*
Manuscript ID	bmjopen-2023-075010.R1
Article Type:	Protocol
Date Submitted by the Author:	04-Nov-2023
Complete List of Authors:	Haque, Rehnuma; International Centre for Diarrhoeal Disease Research Bangladesh, Environmental Interventions Unit; Uppsala Universitet, Dept. of Women’s and Children’s Health<br>Hanson, Molly; Uppsala Universitet, Department of Women's and Children's Health<br>Shariful Islam, Md ; International Centre for Diarrhoeal Disease Research Bangladesh, Environmental Interventions Unit; The University of Queensland, The department of Public Health<br>Akter, Nazrin; International Centre for Diarrhoeal Disease Research Bangladesh, Environmental Interventions Unit<br>Moniruzzaman, Mohammad ; Bangladesh Council of Scientific and Industrial Research<br>Alam, Md. Jahangir; Dhaka Shishu Hospital<br>Kamruzzaman, Md.; Dhaka Shishu Hospital<br>Rahman, Mahbubur; International Centre for Diarrhoeal Disease Research, Environmental Interventions Unit<br>Chisti, Md.; International Centre for Diarrhoeal Disease Research, Bangladesh (icddr,b), Nutrition and Clinical Services Division<br>Raqib, Rubhana; International Centre for Diarrhoeal Disease Research Bangladesh<br>Rahman, Syed; Uppsala University, International Maternal and Child Health, Department of Women's and Children's Health
**Primary Subject Heading**:	Global health
Secondary Subject Heading:	Infectious diseases
Keywords:	Tuberculosis < INFECTIOUS DISEASES, EPIDEMIOLOGIC STUDIES, Community child health < PAEDIATRICS, TOXICOLOGY, Immunology < TROPICAL MEDICINE

SCHOLARONE™
Manuscripts

The pivotal relationship between heavy metal exposures and tuberculosis in Bangladeshi children: Protocol paper of a case-control study

Rehnuma Haque[1,4], Molly Hanson[4], Md. Shariful Islam[5], Nazrin Akter[1], Md. Moniruzzaman[3], Md. Jahangir Alam[2], Md. Kamruzzaman[2], Mahbubur Rahman[1], Mohammod Jobayer Chisti[1], Rubhana Raqib[1], Syed Moshfiqur Rahman[1,4]

1. International Centre for Diarrhoeal Disease Research, Bangladesh, 68 Shahed Tajudin Ahmed Sarani, Mohakhali, Dhaka, Bangladesh, 1212
2. Bangladesh Shishu Hospital & Institute, Sher-e-Bangla Nagar, Dhaka-1207, Bangladesh.
3. Bangladesh Council of Scientific and Industrial Research (BCSIR)
4. Department of Women's and Children's Health, Uppsala University, 752 36 Uppsala, Sweden
5. The School of Public Health, The University of Queensland, Brisbane, Qld, Australia

Corresponding author: Rehnuma Haque, Bangladesh, Dept. of Women's and Children's Health | Uppsala University, +8801755636851, rehnuma.haque@icddrb.org rehnuma.haque@uu.se

Keyword: Tuberculosis, air pollution, heavy metals, Children, LMICs

ABSTRACT

Introduction: Air pollution is a global issue that poses a significant threat to public health. Children, due to their developing physiology, are particularly susceptible to the inhalation of environmental pollutants. Exposure can trigger immune modulation and organ damage, increasing susceptibility to respiratory diseases. Therefore, we aim to examine the association between heavy metal and particulate matter exposure with tuberculosis in children.

Methods and Analysis: As a case-control study, we will include children diagnosed with pulmonary tuberculosis (n=60) and matched healthy controls (n=80) recruited from the same communities in Dhaka, Bangladesh. Exposure data for both cases and controls will be collected by a trained field team conducting home visits. They will administer an exposure questionnaire, measure child anthropometry, collect blood and household dust samples and install 48-hour air quality monitors. The blood samples will be analysed by Inductively Coupled Plasma Mass Spectrometry for serum heavy metal concentrations (Pb, Cd, As, Hg and Cr), as a representative marker of exposure, and the presence of inflammatory biomarkers. Descriptive and inferential statistics, including independent samples t-tests, ANOVA and conditional regression analysis, will be used to quantify heavy metal and particulate matter exposure status in tuberculosis cases compared to healthy controls, while accounting for potential confounders. Dust samples and air quality results will be analysed to understand household sources of heavy metal and particulate matter exposure. To test the study hypothesis, that there is a positive association between exposure and tuberculosis diseases, we will also measure the accumulated effect of simultaneous exposures using Bayesian statistical modelling.

For peer review only - http://bmjopen.bmj.com/site/about/guidelines.xhtml

Article Summary

Strengths and Limitations of this study

- Few case-control studies on a target population of children in highly polluted location.
- Pilot study designed with a small sample size.
- Extensive data collection for a variety of exposures and to control for confounders.
- Unable to differentiate between other sources of exposure, including via food and drinking water.
- Skewed blood metal concentrations will be log-transformed or categorized, potentially resulting in the loss of specific details.

INTRODUCTION

Low- and middle-income countries (LMICs) face the highest burden of both environmental pollution and respiratory infections. The World Health Organization (WHO) estimated globally more than one million children and young adolescents under 15 years fall ill with tuberculosis (TB) every year, and a quarter of those die [1]. An estimated 35,000 children with TB live in Bangladesh. However, determining the underlying causes and preventing TB in children are neglected as global health priorities and remain major challenges for Bangladesh [2]. Similar to many LMICs, Bangladesh is also overburdened by environmental toxicants and pollutants, both naturally occurring and exacerbated by anthropogenic activities. There is growing evidence of high exposure to environmental pollutants in Bangladesh, through drinking water, soil, food, dust and ambient air [3].

Vehicle emissions, spraying pesticides and fertilizers, industrial processes, mining, cooking and combustion are widespread anthropogenic sources of heavy metal pollutants, including arsenic (As), cadmium (Cd), lead (Pb), mercury (Hg) and chromium (Cr) [4, 5]. In Bangladesh, there are health concerns regarding the contamination of common food products and drinking water with heavy metals [5]. In addition, in 2020, AirVisual classified air quality in Bangladesh as 'unhealthy' [6]. Small airborne particle mixtures of heavy metals and organic species, such as carbon compounds, constitute respirable particulate matter (PM) [5, 7]. Living in Dhaka is a significant risk factor for exposure to air pollutants, as the capital city scores almost twenty times higher than the WHO recommended level of PM [6, 7]. Therefore, through overlapping pathways of ingestion, inhalation and dermal absorption, exposure to potentially harmful levels of PM and heavy metals in Bangladesh is inevitable.

Airborne particles of heavy metals and PM cross from the respiratory system into the blood circulation [5, 7]. The mechanisms of toxicity are not fully understood but contribute to inflammation, tissue damage and immune modulation, as described in *figure 1* [8]. Children are particularly susceptible to this environmental pollution toxicity due to their under-developed physiology and immature immune systems [4, 9]. A growing body of research shows that children's lung growth and function is significantly worse in areas with higher air pollution [4, 9]. Simultaneous exposure to multiple environmental pollutants and the interaction between

For peer review only - http://bmjopen.bmj.com/site/about/guidelines.xhtml

heavy metals may also have synergistic effects [8, 10]. Therefore, exposure to PM and heavy metals may alter the immune system, increasing susceptibility to pathogens and respiratory diseases, such as *Mycobacterium tuberculosis* bacteria [9-11].

This paper describes the design of a patient-oriented pilot study which seeks to uncover the association between environmental heavy metals and tuberculosis. The rationale for the proposed study builds on existing research, highlighting that exposure to PM is significantly associated with an increased incidence of pulmonary tuberculosis (PTB) in adults [12]. However, the current body of evidence is limited on the environmental risks for children in Bangladesh. Furthermore, significantly greater progress is required to achieve the UN Sustainable Development Goal, target 3.3, to end tuberculosis epidemics by 2030 [13]. We hypothesise that there is a positive association between PM and heavy metal exposure and PTB diseases in children. This study aims to bridge the current research gap, to understand the role of pollutants in TB pathogenesis, which is crucial to develop appropriate public health interventions and prevention activities.

We will achieve this with the following study objectives, to

(1) test the hypothesis that exposure to heavy metals (e.g., Pb, As, Cd, Hg and Cr) and PM is a risk factor for tuberculosis in children and
(2) quantify environmental sources of heavy metals, and PM exposure among children in urban areas in Dhaka city.

Conceptual framework

Based on existing literature and evidence, this aim reflects a potential causal pathway (*figure 1*) connecting inhalation of airborne pollutants with alterations in the immune system response. Toxic metals constitute ultrafine particulate matter ($PM_{2.5}$), considered the most harmful to the respiratory system, as the particles can deposit in the airways or cross the air-blood barrier in the lungs [8, 10]. In the blood stream, Pb, As, Cd, Hg and Cr accumulate and have been shown to induce toxicity by generating reactive-oxygen species (ROS). ROS suppress the body's antioxidant defence, induce oxidative stress and alter the activation of specific proteins, lipids and enzymes involved in cell defence and the immune response [8]. Toxic metals disrupt immune homeostasis, triggering excessive production of immunoglobulins (IgE and IgA), and pro-inflammatory cytokines and chemokines (TNF-α, IL-1□, IL-6), reduce numbers and/or impair function of immune cells (neutrophils, macrophages, natural killer cells, B and T lymphocytes and their subsets) [10, 14]. These overlapping pathways result in persisting inflammation, immune modulation and organ toxicity.

TB pathogenesis can be attributed to increased susceptibility to infectious diseases and impairment in lung function [8, 10]. Due to their under-developed physiology, children's immature lungs are uniquely susceptible to respiratory tract Infections [4, 10]. Children are crawling, playing and breathing in the air closest to the ground, where high levels of pollutants are concentrated [4]. In contrast to adults, children under five have a faster breathing rate and a relatively immature immune system, making them less efficient in responding to pollutants and pathogens [9].

For peer review only - http://bmjopen.bmj.com/site/about/guidelines.xhtml

Figure 1: Proposed causal pathway, adapted from existing literature [4, 8-11].

METHODS

Study design and setting

We will conduct a case-control study to compare exposure to heavy metals among children diagnosed with TB (case) and matched healthy controls in different urban areas of Dhaka City, Bangladesh.

The population of Dhaka has multiplied over 4 times since 1980, attaining one of the highest population densities in the world, with a remarkable 24 million inhabitants. With persisting urbanisation attracting people to the city, this number is expected to continue growing. As a consequence, rapid urbanisation has aggravated the concentration of pollutants in air, soil and water [15]. Bangladesh, and especially the capital city of Dhaka, has recorded some of the worst air pollution in the world [6]. Existing studies suggest wide-ranging health consequences from exposure to heavy metals and PM through ingestion, inhalation and dermal absorption [3, 5, 7]. Bangladesh was selected as the study site as it shares one of the highest burdens of tuberculosis diseases in the world [2]. The study is hosted by the Environmental Interventions Unit (EIU) of the Infectious Diseases Division, icddr,b.

We will measure whole blood for heavy metal concentrations to assess exposure and conduct immunological assays to evaluate immune status of the patient. We will also conduct household air and dust sampling to identify some sources of heavy metal exposure among children. Household geo-mapping, an exposure questionnaire and socio-economic surveys from the parents will be used to further explore the sources of metal exposure among the children. Written consent will be obtained from guardians/parents and assent from older children (10-14 years) before the data and specimen collection.

Study participants

We will recruit children between the ages of 1 and 14 living in Dhaka city who have been diagnosed with pulmonary tuberculosis (PTB) based on the Bangladesh national TB guideline recommended approach by a registered physician [16]. The national TB guideline recommended a comprehensive approach which includes a careful history taking to identify potential exposure and symptoms suggestive of PTB, a clinical assessment that involves weight monitoring and chest X-rays, bacteriological confirmation through tests like smear microscopy and culture, and additional supportive investigations as needed.

At the beginning of the project, we will visit the TB unit of Dhaka Shishu (Children) Hospital, which is specifically designated for pediatric TB patient's ongoing diagnosis and treatment. To identify eligible participants, we will obtain a registry of TB-diagnosed children, providing the names, ages, addresses and contact details of children diagnosed with PTB. We will then contact the parents or caregivers to request for their child's participation in our study.

Following our inclusion and exclusion criteria, we will enrol 50 children diagnosed with pulmonary TB. In addition, we will then select 50 healthy controls between the ages of 1 and 14 years old who live in the same communities as the cases. According to Rothman et al., it is

For peer review only - http://bmjopen.bmj.com/site/about/guidelines.xhtml

important to match cases with the controls from similar geographic locations as a partial adjustment of environmental and socio-economic factors [17]. The process of selecting controls will be carried out carefully to minimize bias and ensure the validity of our study results. We will identify potential controls by conducting a door-to-door survey within a half-kilometer radius of each case child's residence. We will select the first subsequent child of the same age who participates in shared activities, such as schooling, playing, learning, or mosque visits. To ensure the control children are healthy, the exclusion criteria for controls will be: (i) hospitalisation in the past 60 days, and/or discharged from hospital in the past 30 days; (ii) known chronic disease; (iii) very sick appearance requiring medical attention; (iv) recent cough with blood or painful ear infections; or (v) recovery from diagnosed TB.

To account for potential dropouts, we aim to enrol a total of 60 children in the case group and 80 children in the control group.

Sample size calculation

To determine our required sample size, we have considered the sample size for similar case-control studies with continuous exposures, as described by Lubin et al., [18]. The formula used to calculate sample size was:

$$n = \left(\frac{k+1}{k}\right)\frac{\bar{p}(1-\bar{p})(z_\beta + z_{\alpha/2})^2}{(p_1 - p_2)^2}$$

Where,
k = ratio of control to case,
p_1 = proportion of cases exposed,
p_2 = proportion of controls exposed,
ß = power,
α = level of significance,
$$\bar{p} = \frac{(p_1 + p_2)}{2}$$

We estimate that a sample size of 50 children, in both the case and control groups, would achieve 80% power to detect a mean difference in blood concentrations and odds ratio of 5 at a significance level of 0.05. Recruiting 60 children in the case group and 80 children would allow for a drop out of 10 and 20 children in each group respectively.

Exposure questionnaire and anthropometry
Over a period of six to eight months, a trained field team will conduct home visits and administer a questionnaire to the mother or primary caregiver on household exposures. The questionnaire will cover a range of topics including socioeconomic status (SES), demographics, sources of environmental and household pollution (such as smoking, type of cooking fuel, proximity to battery/textile/waste incineration areas), and other behaviors that may impact pollutant exposure (such as engagement in industrial manufacturing or agricultural practices). Additionally, we will inquire about the children's medical history, including any recent infections or co-existing conditions.

For peer review only

An experienced medical technologist will also gather anthropometric data to assess the child's overall nutritional status. The child's height and weight will be measured using a standardized wooden height scale and digital weight scale (Beurer ps240). These will be validated for accuracy and precision at icddr,b before going to the field.

Clear instructions will be provided to the child prior to weighing, emphasizing the importance of removing any items that could affect the measurement, such as shoes or heavy jackets. The child will be positioned with their weight evenly distributed on both feet, and they will be asked to stand still on the weighing machine with their arms at their sides. The weight measurement will be taken in kilograms and repeated. If the two measurements are noticeably different, we will repeat the process and calculate the average of three measurements to ensure accuracy. Similarly, when measuring height, the child will remove any items that may affect the measurement, such as shoes, socks, headgear, or hair accessories. The child will be informed to stand upright and look straight ahead. Measurements will be taken in centimeters. For infants under 2 years of age, or who cannot stand, we will adjust the anthropometric measurements by laying them on a flat surface for height and in a weighing tray for weight.

Biological sample collection

During the household visits, the field team will also collect blood samples, household dust and indoor air samples, from both case and control groups. These samples will be analyzed to compare biomarkers and environmental exposures between the two groups. By characterizing the sources and risk of pollutant exposure, we can propose intervention strategies to reduce the burden of paediatric TB in low- and middle-income countries.

To collect the blood sample, the area around the cubital vein will first be sterilized. We will use a S-Monovette® Metal analysis Lithium-Heparin 7.5 ml tube and Safety-Multifly® needle to draw 5ml of blood from the child's vein. Each sample tube will be carefully labelled with a unique barcode and anonymous relevant information, including the date of collection and household ID. After collecting the blood sample tubes will be transported in cool box to the Immunobiology, Nutrition and Toxicology Laboratory at icddr,b for analysis. 1 ml of whole blood will be removed into cryovials, and the remaining blood will be centrifuged to separate plasma from red blood cell components. All aliquots will be stored at -80◦C.

For heavy metal concentrations in blood/plasma, the primary exposures of interest are lead (Pb) and cadmium (Cd), while secondary exposures include arsenic (As), mercury (Hg) and chromium (Cr). Aliquots of blood will be analysed for these metals by Inductively Coupled Plasma Mass Spectrometry (ICP-MS). As we will conduct ICP-MS analysis therefore we will get the panel of heavy metal result at once. The immunoglobulins, pro-inflammatory cytokines and chemokines will be tested in plasma by enzyme-linked immunosorbent assay (ELISA) or automated immune analyser as appropriate. The leftover samples (whole blood and plasma) will be preserved for future analysis of inflammatory and nutritional markers in relation to environmental exposures mostly heavy metals complying with the icddr,b specimen storage policy, for relevant analysis depending on the future availability of funds.

For peer review only - http://bmjopen.bmj.com/site/about/guidelines.xhtml

Environmental sample collection

We will install portable air quality monitors (PurpleAir) in participants' homes to measure real-time $PM_{2.5}$ levels for at least 48 hours. These will be placed in the child's bedroom or other places where they spend most of their time at home. Data on $PM_{2.5}$, humidity, and temperature will be sent directly to our smart devices.

During the household visits, the team will collect samples of household dust from the children's homes. These will be analysed at the Environmental Interventions Unit, icddr,b using x-ray fluorescence spectrometry (XRF). A subset of dust samples will be sent to the Bangladesh Council of Scientific and Industrial Research (BCSIR) laboratory for quality assurance, where repeat measures will be performed by ICP-MS and data will be compared with XRF data. The results will be used to fulfil the second research objective, to identify and quantify environmental sources of heavy metals and PM.

Data management and analysis plan

A data management standard operating protocol will be developed considering the range and variation in data types. Field researcher officers will consult with the research assistants on a daily basis to check data consistency. The household questionnaire data will be digitally programmed through tablet computers, downloaded from the devices each week and reviewed by an Environmental Interventions Unit (EIU) statistician. The statistician will update the server and produce datasets for the investigators in R programme language. Once the data are available to the investigators, they will run previously developed Stata do files to assess inconsistencies and send queries to the field team. All metadata will be stored on the EIU's Data Repository System and a backup will be taken.

As evidenced in similar studies, the distributions of Pb, As, Cd, Hg and Cr in the blood samples are expected to be skewed. In order to fulfil the assumptions of the selected parametric tests, this data will be transformed, by either natural log transformation or categorisation of the metal concentrations.

Descriptive analysis, including frequency tests, will be stratified by child's age, sex and TB condition. Chi-squared, independent samples t-tests or ANOVA will be performed to compare the blood heavy metal concentrations among TB cases and healthy controls. In addition, we will compare the sociodemographic information, based on the parental exposure questionnaire survey. We will use conditional regression analysis to estimate the biomarkers that are associated with heavy metal and PM exposures among TB children. In addition, if we identify two or more elevated metals in the blood and/or environmental samples, we will measure their accumulated effect using Bayesian statistical modelling [19].

Ethical considerations

The study design and protocols for data collection will be submitted for review by the Institutional Review Board (IRB) of icddr,b consisting of the Research Review Committee (RRC) and the Ethical Review Committee (ERC). The study will be initiated after it has been approved by the IRB. The investigators who will be managing and analyzing the human data will have up-to-date ethics certification.

For peer review only - http://bmjopen.bmj.com/site/about/guidelines.xhtml

There are minor risks to the participants during sample collection. The risks and advantages for participants will be provided in written information forms to ensure the participants are fully informed about the study. The purpose and methods of the study will also be clearly described. This information will be written in English and translated into Bangla in a format that is easy to understand even with little or no educational background. If required, the form will also be read out to the participant.

As this study recruits children between one and 15 years of age, the mothers or caregivers will be required to give consent on behalf of their children participating in the study. Before taking their consent, the researchers will repeat the study information and participants will have time to ask any questions. The participants will also be informed that they have the freedom to withdraw from the study at any time and have no adverse implications. Those who agree to participate will be required to give written informed consent, by signing or making an imprint of their left thumb and verified by an independent witness. Participants will also keep a copy of the information and consent forms so they have open access and can contact the research review committee if necessary.

Field data and the sample test results will be uploaded to the secured server at icddr,b. Only investigators will have access to the dataset. Prior to uploading the data, the field supervisor will check the datasets for consistency and identify any missing information. If any inconsistencies are identified, queries will be sent to the field and resolution of the discrepancies will be made. Throughout the data management, confidentiality of information will be strictly maintained, and access to the data will be restricted to the research team members only. At the time of analyzing information and publishing the results of the study, the name or identity of the study participants will not be used.

Patient and Public Involvement

Patients were not involved to develop this protocol paper.

Expected results

The hypothesis-driven research we propose is intended to address the gap in developing childhood TB diseases and the association with environmental pollutant exposure, with a focus on air pollution and heavy metal exposure. This research will provide evidence for follow-up studies to improve understanding of the causal pathway connecting these pollutants to TB diseases and inform evidence-based guidance to eliminate TB. Our long-term goal is to evaluate the chemical risk factors for respiratory infections and to improve children's health.

Declaration of competing interest

The authors declare that they have no known competing financial interests or personal relationships that could have appeared to influence the work reported in this paper.

Acknowledgements

We express our gratitude to the governing body for granting us permission to carry out patient enrollment at Dhaka Shishu Hospital and access to laboratory facilities at Bangladesh Council of Scientific and Industrial Research (BCSIR) for our research endeavors. icddr, b gratefully

For peer review only - http://bmjopen.bmj.com/site/about/guidelines.xhtml

acknowledges our core donors including the Governments of Bangladesh and Canada for their support and commitment to icddr, b research efforts.

Funding

This research is funded by Thrasher Research Fund- Early Career Award number 01477.

Contributors

RH conceptualized the study, drafted the original manuscript and secured the funding. MH, MSI and SMR supported in the first draft of manuscript development. RH, NA, MR, MJC, RR, MJA and MK provided input in study design and site selection. RR and MM provided support in laboratory SOP development. All authors participated in editing, reviewing, and approved the final version of the manuscript for publication.

REFERENCES

[1] World Health Organization, "'WHO Consolidated Guidelines on Tuberculosis: Module 5: Management of Tuberculosis in Children and Adolescents'," 2022. [Online]. Available: https://www.who.int/publications-detail-redirect/9789240046764.

[2] N. Kak, K. Chakraborty, S. Sadaphal, H. J. AlMossawi, M. Calnan, and B. Vikarunnessa, "Strategic priorities for TB control in Bangladesh, Indonesia, and the Philippines - comparative analysis of national TB prevalence surveys," (in eng), *BMC Public Health,* vol. 20, no. 1, p. 560, Apr 25 2020, doi: 10.1186/s12889-020-08675-9.

[3] M. M. Islam, M. R. Karim, X. Zheng, and X. Li, "Heavy Metal and Metalloid Pollution of Soil, Water and Foods in Bangladesh: A Critical Review," (in eng), *Int J Environ Res Public Health,* vol. 15, no. 12, Dec 11 2018, doi: 10.3390/ijerph15122825.

[4] World Health Organization, "Air Pollution and Child Health: Prescribing Clean Air'. Summary," Geneva, 2018.

[5] A. Sarker *et al.*, "Heavy metals contamination and associated health risks in food webs—a review focuses on food safety and environmental sustainability in Bangladesh," *Environmental Science and Pollution Research,* vol. 29, no. 3, pp. 3230-3245, 2022.

[6] IQAir., "Dhaka Air Quality Index (AQI)'. Air Quality Index (AQI) and PM2.5 Air Pollution in Dhaka.," 1 February 2023 2023. [Online]. Available: https://www.iqair.com/bangladesh/dhaka.

[7] M. R. S. Pavel, M. Tarannum, S. U. Zaman, F. Jeba, and A. Salam, "Health Risk Assessment of High-Level Particulate Matter Exposure in Different Environments in Mega City Dhaka, Bangladesh," *Bangl. J. Sci. Res.,* vol. 2, pp. 78-87, 2020.

[8] M. Balali-Mood, K. Naseri, Z. Tahergorabi, M. R. Khazdair, and M. Sadeghi, "Toxic Mechanisms of Five Heavy Metals: Mercury, Lead, Chromium, Cadmium, and Arsenic," (in eng), *Front Pharmacol,* vol. 12, p. 643972, 2021, doi: 10.3389/fphar.2021.643972.

[9] P. J. Landrigan *et al.*, "Pollution and children's health," (in eng), *Sci Total Environ,* vol. 650, no. Pt 2, pp. 2389-2394, Feb 10 2019, doi: 10.1016/j.scitotenv.2018.09.375.

[10] M. L. Ackland *et al.*, "Metals in the Environment as Risk Factors for Infectious Diseases: Gaps and Opportunities," in *Trace Metals and Infectious Diseases*, J. O. Nriagu and E. P. Skaar Eds. Cambridge (MA): MIT Press, 2015.

For peer review only - http://bmjopen.bmj.com/site/about/guidelines.xhtml

[11] J. L. Davis and W. Checkley, "Characterization of Air Pollution Exposures as Risk Factors for Tuberculosis Infection," (in eng), *Am J Respir Crit Care Med,* vol. 204, no. 10, pp. 1130-1131, Nov 15 2021, doi: 10.1164/rccm.202107-1795ED.

[12] C. A. Dimala and B. M. Kadia, "A systematic review and meta-analysis on the association between ambient air pollution and pulmonary tuberculosis," (in eng), *Sci Rep,* vol. 12, no. 1, p. 11282, Jul 4 2022, doi: 10.1038/s41598-022-15443-9.

[13] United Nations Statistical Division, "3.3.2 Tuberculosis Incidence'," Global SDG Indicator Platform, 31 October 2018. 2018. [Online]. Available: https://sdg.tracking-progress.org/indicator/3-3-2-tuberculosis-incidence/.

[14] A. U. Anka *et al.*, "Potential mechanisms of some selected heavy metals in the induction of inflammation and autoimmunity," *European Journal of Inflammation,* vol. 20, p. 1721727X221122719, 2022/09/01 2022, doi: 10.1177/1721727X221122719.

[15] J. A. L. Bird, Yue%A Rahman, Hossain Zillur%A Rama, Martin%A Venables, Anthony J., *Toward Great Dhaka: A New Urban Development Paradigm Eastward* (Toward Great Dhaka: A New Urban Development Paradigm Eastward).

[16] NTP, "National Guideline and Operational Manual for Tuberculosis," Dhaka, Bangladesh., 2021.

[17] K. J. Rothman, S. Greenland, and T. L. Lash, *Modern epidemiology*. Wolters Kluwer Health/Lippincott Williams & Wilkins Philadelphia, 2008.

[18] J. H. Lubin, M. H. Gail, and A. G. Ershow, "Sample size and power for case-control studies when exposures are continuous," (in eng), *Stat Med,* vol. 7, no. 3, pp. 363-76, Mar 1988, doi: 10.1002/sim.4780070302.

[19] S. Shah-Kulkarni *et al.*, "Prenatal exposure to mixtures of heavy metals and neurodevelopment in infants at 6 months," *Environmental research,* vol. 182, p. 109122, 2020.

Figure 1: Proposed causal pathway, adapted from existing literature [4, 8-11].

For peer review only - http://bmjopen.bmj.com/site/about/guidelines.xhtml

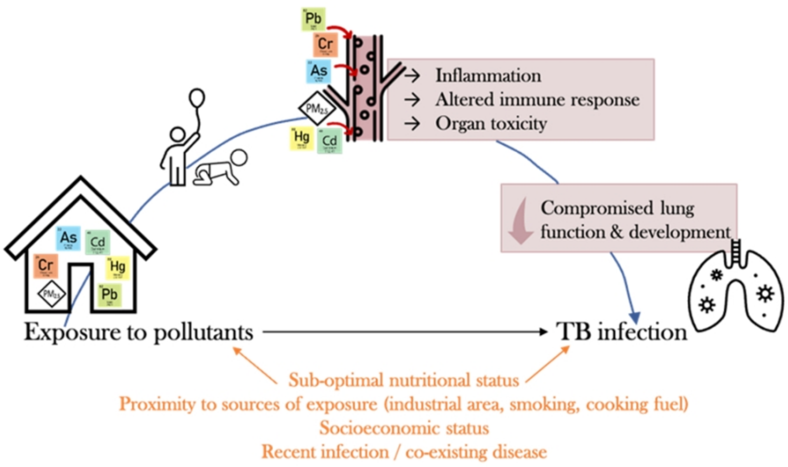

Figure 1: Proposed causal pathway, adapted from existing literature [4, 8-11].

855x481mm (38 x 38 DPI)

BMJ Open

The pivotal relationship between heavy metal exposures and tuberculosis in Bangladeshi children: Protocol paper of a case-control study

Journal:	*BMJ Open*
Manuscript ID	bmjopen-2023-075010.R2
Article Type:	Protocol
Date Submitted by the Author:	30-Nov-2023

Complete List of Authors:	Haque, Rehnuma; International Centre for Diarrhoeal Disease Research Bangladesh, Environmental Health and WASH, Health System Population and Studies Division; Uppsala Universitet, Department of Women's and Children's Health, Global Health and Migration<br>Hanson, Molly; Uppsala Universitet, Department of Women's and Children's Health, Global Health and Migration<br>Shariful Islam, Md ; International Centre for Diarrhoeal Disease Research Bangladesh, Environmental Health and WASH, Health System Population and Studies Division; The University of Queensland, The department of Public Health<br>Akter, Nazrin; International Centre for Diarrhoeal Disease Research Bangladesh, Environmental Health and WASH, Health System Population and Studies Division<br>Moniruzzaman, Mohammad ; Bangladesh Council of Scientific and Industrial Research<br>Alam, Md. Jahangir; Dhaka Shishu Hospital<br>Kamruzzaman, Md.; Dhaka Shishu Hospital<br>Rahman, Mahbubur; International Centre for Diarrhoeal Disease Research Bangladesh, Environmental Health and WASH, Health System Population and Studies Division; Uppsala Universitet, Department of Women's and Children's Health, Global Health and Migration<br>Chisti, Md.; International Centre for Diarrhoeal Disease Research Bangladesh, Nutrition Research Division<br>Raqib, Rubhana; International Centre for Diarrhoeal Disease Research Bangladesh, Immunobiology Nutrition and Toxicology Laboratory, Nutrition Research Division<br>Rahman, Syed; Uppsala Universitet, Department of Women's and Children's Health, Global Health and Migration

**Primary Subject Heading**:	Global health
Secondary Subject Heading:	Infectious diseases
Keywords:	Tuberculosis < INFECTIOUS DISEASES, EPIDEMIOLOGIC STUDIES, Community child health < PAEDIATRICS, TOXICOLOGY, Immunology < TROPICAL MEDICINE

The pivotal relationship between heavy metal exposures and tuberculosis in Bangladeshi children: Protocol paper of a case-control study

Rehnuma Haque[1,4], Molly Hanson[4], Md. Shariful Islam[5], Nazrin Akter[1], Md. Moniruzzaman[3], Md. Jahangir Alam[2], Md. Kamruzzaman[2], Mahbubur Rahman[1], Mohammod Jobayer Chisti[1], Rubhana Raqib[1], Syed Moshfiqur Rahman[1,4]

1. International Centre for Diarrhoeal Disease Research, Bangladesh, 68 Shahed Tajudin Ahmed Sarani, Mohakhali, Dhaka, Bangladesh, 1212
2. Bangladesh Shishu Hospital & Institute, Sher-e-Bangla Nagar, Dhaka-1207, Bangladesh.
3. Bangladesh Council of Scientific and Industrial Research (BCSIR)
4. Department of Women's and Children's Health, Uppsala University, 752 36 Uppsala, Sweden
5. The School of Public Health, The University of Queensland, Brisbane, Qld, Australia

Corresponding author: Rehnuma Haque, Bangladesh, Dept. of Women's and Children's Health | Uppsala University, +8801755636851, rehnuma.haque@icddrb.org rehnuma.haque@uu.se

Keyword: Tuberculosis, air pollution, heavy metals, Children, LMICs

ABSTRACT

Introduction: Air pollution is a global issue that poses a significant threat to public health. Children, due to their developing physiology, are particularly susceptible to the inhalation of environmental pollutants. Exposure can trigger immune modulation and organ damage, increasing susceptibility to respiratory diseases. Therefore, we aim to examine the association between heavy metal and particulate matter exposure with tuberculosis in children.

Methods and Analysis: As a case-control study, we will include children diagnosed with pulmonary tuberculosis (n=60) and matched healthy controls (n=80) recruited from the same communities in Dhaka, Bangladesh. Exposure data for both cases and controls will be collected by a trained field team conducting home visits. They will administer an exposure questionnaire, measure child anthropometry, collect blood and household dust samples and install 48-hour air quality monitors. The blood samples will be analysed by Inductively Coupled Plasma Mass Spectrometry for serum heavy metal concentrations (Pb, Cd, As, Hg and Cr), as a representative marker of exposure, and the presence of inflammatory biomarkers. Descriptive and inferential statistics, including independent samples t-tests, ANOVA and conditional regression analysis, will be used to quantify heavy metal and particulate matter exposure status in tuberculosis cases compared to healthy controls, while accounting for potential confounders. Dust samples and air quality results will be analysed to understand household sources of heavy metal and particulate matter exposure. To test the study hypothesis, that there is a positive association between exposure and tuberculosis diseases, we will also measure the accumulated effect of simultaneous exposures using Bayesian statistical modelling.

Ethics and dissemination: This study has been approved by International Centre for Diarrhoeal Disease Research, Bangladesh's Institutional Review Board (PR-22030). The study findings will be

For peer review only - http://bmjopen.bmj.com/site/about/guidelines.xhtml

For peer review only

disseminated at conferences and published in peer-reviewed journals.

Article Summary

Strengths and Limitations of this study

- Few case-control studies on a target population of children in highly polluted location.
- Pilot study designed with a small sample size.
- Extensive data collection for a variety of exposures and to control for confounders.
- Unable to differentiate between other sources of exposure, including via food and drinking water.
- Skewed blood metal concentrations will be log-transformed or categorized, potentially resulting in the loss of specific details.

INTRODUCTION

Low- and middle-income countries (LMICs) face the highest burden of both environmental pollution and respiratory infections. The World Health Organization (WHO) estimated globally more than one million children and young adolescents under 15 years fall ill with tuberculosis (TB) every year, and a quarter of those die [1]. An estimated 35,000 children with TB live in Bangladesh. However, determining the underlying causes and preventing TB in children are neglected as global health priorities and remain major challenges for Bangladesh [2]. Similar to many LMICs, Bangladesh is also overburdened by environmental toxicants and pollutants, both naturally occurring and exacerbated by anthropogenic activities. There is growing evidence of high exposure to environmental pollutants in Bangladesh, through drinking water, soil, food, dust and ambient air [3].

Vehicle emissions, spraying pesticides and fertilizers, industrial processes, mining, cooking and combustion are widespread anthropogenic sources of heavy metal pollutants, including arsenic (As), cadmium (Cd), lead (Pb), mercury (Hg) and chromium (Cr) [4, 5]. In Bangladesh, there are health concerns regarding the contamination of common food products and drinking water with heavy metals [5]. In addition, in 2020, AirVisual classified air quality in Bangladesh as 'unhealthy' [6]. Small airborne particle mixtures of heavy metals and organic species, such as carbon compounds, constitute respirable particulate matter (PM) [5, 7]. Living in Dhaka is a significant risk factor for exposure to air pollutants, as the capital city scores almost twenty times higher than the WHO recommended level of PM [6, 7]. Therefore, through overlapping pathways of ingestion, inhalation and dermal absorption, exposure to potentially harmful levels of PM and heavy metals in Bangladesh is inevitable.

Airborne particles of heavy metals and PM cross from the respiratory system into the blood circulation [5, 7]. The mechanisms of toxicity are not fully understood but contribute to inflammation, tissue damage and immune modulation, as described in *figure 1* [8]. Children are particularly susceptible to this environmental pollution toxicity due to their under-developed physiology and immature immune systems [4, 9]. A growing body of research shows that children's lung growth and function is significantly worse in areas with higher air pollution [4, 9]. Simultaneous exposure to multiple environmental pollutants and the interaction between heavy metals may also have synergistic effects [8, 10]. Therefore, exposure to PM and heavy

For peer review only - http://bmjopen.bmj.com/site/about/guidelines.xhtml

metals may alter the immune system, increasing susceptibility to pathogens and respiratory diseases, such as *Mycobacterium tuberculosis* bacteria [9-11].

This paper describes the design of a patient-oriented pilot study which seeks to uncover the association between environmental heavy metals and tuberculosis. The rationale for the proposed study builds on existing research, highlighting that exposure to PM is significantly associated with an increased incidence of pulmonary tuberculosis (PTB) in adults [12]. However, the current body of evidence is limited on the environmental risks for children in Bangladesh. Furthermore, significantly greater progress is required to achieve the UN Sustainable Development Goal, target 3.3, to end tuberculosis epidemics by 2030 [13]. We hypothesise that there is a positive association between PM and heavy metal exposure and PTB diseases in children. This study aims to bridge the current research gap, to understand the role of pollutants in TB pathogenesis, which is crucial to develop appropriate public health interventions and prevention activities.

We will achieve this with the following study objectives, to

(1) test the hypothesis that exposure to heavy metals (e.g., Pb, As, Cd, Hg and Cr) and PM is a risk factor for tuberculosis in children and
(2) quantify environmental sources of heavy metals, and PM exposure among children in urban areas in Dhaka city.

Conceptual framework

Based on existing literature and evidence, this aim reflects a potential causal pathway (*figure 1*) connecting inhalation of airborne pollutants with alterations in the immune system response. Toxic metals constitute ultrafine particulate matter ($PM_{2.5}$), considered the most harmful to the respiratory system, as the particles can deposit in the airways or cross the air-blood barrier in the lungs [8, 10]. In the blood stream, Pb, As, Cd, Hg and Cr accumulate and have been shown to induce toxicity by generating reactive-oxygen species (ROS). ROS suppress the body's antioxidant defence, induce oxidative stress and alter the activation of specific proteins, lipids and enzymes involved in cell defence and the immune response [8]. Toxic metals disrupt immune homeostasis, triggering excessive production of immunoglobulins (IgE and IgA), and pro-inflammatory cytokines and chemokines (TNF-α, IL-1□, IL-6), reduce numbers and/or impair function of immune cells (neutrophils, macrophages, natural killer cells, B and T lymphocytes and their subsets) [10, 14]. These overlapping pathways result in persisting inflammation, immune modulation and organ toxicity.

TB pathogenesis can be attributed to increased susceptibility to infectious diseases and impairment in lung function [8, 10]. Due to their under-developed physiology, children's immature lungs are uniquely susceptible to respiratory tract Infections [4, 10]. Children are crawling, playing and breathing in the air closest to the ground, where high levels of pollutants are concentrated [4]. In contrast to adults, children under five have a faster breathing rate and a relatively immature immune system, making them less efficient in responding to pollutants and pathogens [9].

Figure 1: Proposed causal pathway, adapted from existing literature [4, 8-11].

For peer review only

For peer review only - http://bmjopen.bmj.com/site/about/guidelines.xhtml

METHODS

Study design and setting

We will conduct a case-control study to compare exposure to heavy metals among children diagnosed with TB (case) and matched healthy controls in different urban areas of Dhaka City, Bangladesh.

The population of Dhaka has multiplied over 4 times since 1980, attaining one of the highest population densities in the world, with a remarkable 24 million inhabitants. With persisting urbanisation attracting people to the city, this number is expected to continue growing. As a consequence, rapid urbanisation has aggravated the concentration of pollutants in air, soil and water [15]. Bangladesh, and especially the capital city of Dhaka, has recorded some of the worst air pollution in the world [6]. Existing studies suggest wide-ranging health consequences from exposure to heavy metals and PM through ingestion, inhalation and dermal absorption [3, 5, 7]. Bangladesh was selected as the study site as it shares one of the highest burdens of tuberculosis diseases in the world [2]. The study is hosted by the Environmental Interventions Unit (EIU) of the Infectious Diseases Division, icddr,b.

We will measure whole blood for heavy metal concentrations to assess exposure and conduct immunological assays to evaluate immune status of the patient. We will also conduct household air and dust sampling to identify some sources of heavy metal exposure among children. Household geo-mapping, an exposure questionnaire and socio-economic surveys from the parents will be used to further explore the sources of metal exposure among the children. Written consent will be obtained from guardians/parents and assent from older children (10-14 years) before the data and specimen collection.

Study participants

We will recruit children between the ages of 1 and 14 living in Dhaka city who have been diagnosed with pulmonary tuberculosis (PTB) based on the Bangladesh national TB guideline recommended approach by a registered physician [16]. The national TB guideline recommended a comprehensive approach which includes a careful history taking to identify potential exposure and symptoms suggestive of PTB, a clinical assessment that involves weight monitoring and chest X-rays, bacteriological confirmation through tests like smear microscopy and culture, and additional supportive investigations as needed.

At the beginning of the project, we will visit the TB unit of Dhaka Shishu (Children) Hospital, which is specifically designated for pediatric TB patient's ongoing diagnosis and treatment. To identify eligible participants, we will obtain a registry of TB-diagnosed children, providing the names, ages, addresses and contact details of children diagnosed with PTB. We will then contact the parents or caregivers to request for their child's participation in our study.

Following our inclusion and exclusion criteria, we will enrol 50 children diagnosed with pulmonary TB. In addition, we will then select 50 healthy controls between the ages of 1 and 14 years old who live in the same communities as the cases. According to Rothman et al., it is important to match cases with the controls from similar geographic locations as a partial

For peer review only - http://bmjopen.bmj.com/site/about/guidelines.xhtml

adjustment of environmental and socio-economic factors [17]. The process of selecting controls will be carried out carefully to minimize bias and ensure the validity of our study results. We will identify potential controls by conducting a door-to-door survey within a half-kilometer radius of each case child's residence. We will select the first subsequent child of the same age who participates in shared activities, such as schooling, playing, learning, or mosque visits. To ensure the control children are healthy, the exclusion criteria for controls will be: (i) hospitalisation in the past 60 days, and/or discharged from hospital in the past 30 days; (ii) known chronic disease; (iii) very sick appearance requiring medical attention; (iv) recent cough with blood or painful ear infections; or (v) recovery from diagnosed TB.

To account for potential dropouts, we aim to enrol a total of 60 children in the case group and 80 children in the control group.

Sample size calculation

To determine our required sample size, we have considered the sample size for similar case-control studies with continuous exposures, as described by Lubin et al., [18]. The formula used to calculate sample size was:

$$n = \left(\frac{k+1}{k}\right)\frac{\overline{p}(1-\overline{p})(z_\beta + z_{\alpha/2})^2}{(p_1 - p_2)^2}$$

Where,
k = ratio of control to case,
p_1 = proportion of cases exposed,
p_2 = proportion of controls exposed,
ß = power,
α = level of significance,

$$\overline{p} = \frac{(p_1 + p_2)}{2}$$

We estimate that a sample size of 50 children, in both the case and control groups, would achieve 80% power to detect a mean difference in blood concentrations and odds ratio of 5 at a significance level of 0.05. Recruiting 60 children in the case group and 80 children would allow for a drop out of 10 and 20 children in each group respectively.

Exposure questionnaire and anthropometry

Over a period of six to eight months, a trained field team will conduct home visits and administer a questionnaire to the mother or primary caregiver on household exposures. The questionnaire will cover a range of topics including socioeconomic status (SES), demographics, sources of environmental and household pollution (such as smoking, type of cooking fuel, proximity to battery/textile/waste incineration areas), and other behaviors that may impact pollutant exposure (such as engagement in industrial manufacturing or agricultural practices). Additionally, we will inquire about the children's medical history, including any recent infections or co-existing conditions.

For peer review only - http://bmjopen.bmj.com/site/about/guidelines.xhtml

An experienced medical technologist will also gather anthropometric data to assess the child's overall nutritional status. The child's height and weight will be measured using a standardized wooden height scale and digital weight scale (Beurer ps240). These will be validated for accuracy and precision at icddr,b before going to the field.

Clear instructions will be provided to the child prior to weighing, emphasizing the importance of removing any items that could affect the measurement, such as shoes or heavy jackets. The child will be positioned with their weight evenly distributed on both feet, and they will be asked to stand still on the weighing machine with their arms at their sides. The weight measurement will be taken in kilograms and repeated. If the two measurements are noticeably different, we will repeat the process and calculate the average of three measurements to ensure accuracy. Similarly, when measuring height, the child will remove any items that may affect the measurement, such as shoes, socks, headgear, or hair accessories. The child will be informed to stand upright and look straight ahead. Measurements will be taken in centimeters. For infants under 2 years of age, or who cannot stand, we will adjust the anthropometric measurements by laying them on a flat surface for height and in a weighing tray for weight.

Biological sample collection

During the household visits, the field team will also collect blood samples, household dust and indoor air samples, from both case and control groups. These samples will be analyzed to compare biomarkers and environmental exposures between the two groups. By characterizing the sources and risk of pollutant exposure, we can propose intervention strategies to reduce the burden of paediatric TB in low- and middle-income countries.

To collect the blood sample, the area around the cubital vein will first be sterilized. We will use a S-Monovette® Metal analysis Lithium-Heparin 7.5 ml tube and Safety-Multifly® needle to draw 5ml of blood from the child's vein. Each sample tube will be carefully labelled with a unique barcode and anonymous relevant information, including the date of collection and household ID. After collecting the blood sample tubes will be transported in cool box to the Immunobiology, Nutrition and Toxicology Laboratory at icddr,b for analysis. 1 ml of whole blood will be removed into cryovials, and the remaining blood will be centrifuged to separate plasma from red blood cell components. All aliquots will be stored at -80◦C.

For heavy metal concentrations in blood/plasma, the primary exposures of interest are lead (Pb) and cadmium (Cd), while secondary exposures include arsenic (As), mercury (Hg) and chromium (Cr). Aliquots of blood will be analysed for these metals by Inductively Coupled Plasma Mass Spectrometry (ICP-MS). As we will conduct ICP-MS analysis therefore we will get the panel of heavy metal result at once. The immunoglobulins, pro-inflammatory cytokines and chemokines will be tested in plasma by enzyme-linked immunosorbent assay (ELISA) or automated immune analyser as appropriate. The leftover samples (whole blood and plasma) will be preserved for future analysis of inflammatory and nutritional markers in relation to environmental exposures mostly heavy metals complying with the icddr,b specimen storage policy, for relevant analysis depending on the future availability of funds.

Environmental sample collection

For peer review only - http://bmjopen.bmj.com/site/about/guidelines.xhtml

We will install portable air quality monitors (PurpleAir) in participants' homes to measure real-time $PM_{2.5}$ levels for at least 48 hours. These will be placed in the child's bedroom or other places where they spend most of their time at home. Data on $PM_{2.5}$, humidity, and temperature will be sent directly to our smart devices.

During the household visits, the team will collect samples of household dust from the children's homes. These will be analysed at the Environmental Interventions Unit, icddr,b using x-ray fluorescence spectrometry (XRF). A subset of dust samples will be sent to the Bangladesh Council of Scientific and Industrial Research (BCSIR) laboratory for quality assurance, where repeat measures will be performed by ICP-MS and data will be compared with XRF data. The results will be used to fulfil the second research objective, to identify and quantify environmental sources of heavy metals and PM.

Data management and analysis plan

A data management standard operating protocol will be developed considering the range and variation in data types. Field researcher officers will consult with the research assistants on a daily basis to check data consistency. The household questionnaire data will be digitally programmed through tablet computers, downloaded from the devices each week and reviewed by an Environmental Interventions Unit (EIU) statistician. The statistician will update the server and produce datasets for the investigators in R programme language. Once the data are available to the investigators, they will run previously developed Stata do files to assess inconsistencies and send queries to the field team. All metadata will be stored on the EIU's Data Repository System and a backup will be taken.

As evidenced in similar studies, the distributions of Pb, As, Cd, Hg and Cr in the blood samples are expected to be skewed. In order to fulfil the assumptions of the selected parametric tests, this data will be transformed, by either natural log transformation or categorisation of the metal concentrations.

Descriptive analysis, including frequency tests, will be stratified by child's age, sex and TB condition. Chi-squared, independent samples t-tests or ANOVA will be performed to compare the blood heavy metal concentrations among TB cases and healthy controls. In addition, we will compare the sociodemographic information, based on the parental exposure questionnaire survey. We will use conditional regression analysis to estimate the biomarkers that are associated with heavy metal and PM exposures among TB children. In addition, if we identify two or more elevated metals in the blood and/or environmental samples, we will measure their accumulated effect using Bayesian statistical modelling [19].

Ethics and dissemination

The study design and protocols have received approval from the Institutional Review Board (PR-22030) of the International Centre for Diarrhoeal Disease Research, Bangladesh (icddrb). This board comprises the Research Review Committee (RRC) and the Ethical Review Committee (ERC). Field activities and sample collection for the study commenced on June 1, 2023 and will continue until May 25 2024.

For peer review only

For peer review only - http://bmjopen.bmj.com/site/about/guidelines.xhtml

There are minor risks to the participants during sample collection. The risks and advantages for participants will be provided in written information forms to ensure the participants are fully informed about the study. The purpose and methods of the study will also be clearly described. This information will be written in English and translated into Bangla in a format that is easy to understand even with little or no educational background. If required, the form will also be read out to the participant.

As this study recruits children between one and 15 years of age, the mothers or caregivers will be required to give consent on behalf of their children participating in the study. Before taking their consent, the researchers will repeat the study information and participants will have time to ask any questions. The participants will also be informed that they have the freedom to withdraw from the study at any time and have no adverse implications. Those who agree to participate will be required to give written informed consent, by signing or making an imprint of their left thumb and verified by an independent witness. Participants will also keep a copy of the information and consent forms so they have open access and can contact the research review committee if necessary.

Field data and the sample test results will be uploaded to the secured server at icddr,b. Only investigators will have access to the dataset. Prior to uploading the data, the field supervisor will check the datasets for consistency and identify any missing information. If any inconsistencies are identified, queries will be sent to the field and resolution of the discrepancies will be made. Throughout the data management, confidentiality of information will be strictly maintained, and access to the data will be restricted to the research team members only. At the time of analyzing information and publishing the results of the study, the name or identity of the study participants will not be used.

The study results will be shared within icddrb through scientific seminars and the annual report. Additionally, we will disseminate the findings to our collaborators and participate in national and international conferences. Also, the results will be published in peer-reviewed journals

Patient and Public Involvement

Patients were not involved to develop this protocol paper.

Expected results

The hypothesis-driven research we propose is intended to address the gap in developing childhood TB diseases and the association with environmental pollutant exposure, with a focus on air pollution and heavy metal exposure. This research will provide evidence for follow-up studies to improve understanding of the causal pathway connecting these pollutants to TB diseases and inform evidence-based guidance to eliminate TB. Our long-term goal is to evaluate the chemical risk factors for respiratory infections and to improve children's health.

Declaration of competing interest

The authors declare that they have no known competing financial interests or personal relationships that could have appeared to influence the work reported in this paper.

Acknowledgements

For peer review only

For peer review only - http://bmjopen.bmj.com/site/about/guidelines.xhtml

We express our gratitude to the governing body for granting us permission to carry out patient enrollment at Dhaka Shishu Hospital and access to laboratory facilities at Bangladesh Council of Scientific and Industrial Research (BCSIR) for our research endeavors. icddr, b gratefully acknowledges our core donors including the Governments of Bangladesh and Canada for their support and commitment to icddr, b research efforts.

Funding

This research is funded by Thrasher Research Fund- Early Career Award number 01477.

Contributors

RH conceptualized the study, drafted the original manuscript and secured the funding. MH, MSI and SMR supported in the first draft of manuscript development. RH, NA, MR, MJC, RR, MJA and MK provided input in study design and site selection. RR and MM provided support in laboratory SOP development. All authors participated in editing, reviewing, and approved the final version of the manuscript for publication.

REFERENCES

[1] World Health Organization, "'WHO Consolidated Guidelines on Tuberculosis: Module 5: Management of Tuberculosis in Children and Adolescents'," 2022. [Online]. Available: https://www.who.int/publications-detail-redirect/9789240046764.

[2] N. Kak, K. Chakraborty, S. Sadaphal, H. J. AlMossawi, M. Calnan, and B. Vikarunnessa, "Strategic priorities for TB control in Bangladesh, Indonesia, and the Philippines - comparative analysis of national TB prevalence surveys," (in eng), *BMC Public Health,* vol. 20, no. 1, p. 560, Apr 25 2020, doi: 10.1186/s12889-020-08675-9.

[3] M. M. Islam, M. R. Karim, X. Zheng, and X. Li, "Heavy Metal and Metalloid Pollution of Soil, Water and Foods in Bangladesh: A Critical Review," (in eng), *Int J Environ Res Public Health,* vol. 15, no. 12, Dec 11 2018, doi: 10.3390/ijerph15122825.

[4] World Health Organization, "Air Pollution and Child Health: Prescribing Clean Air'. Summary," Geneva, 2018.

[5] A. Sarker *et al.*, "Heavy metals contamination and associated health risks in food webs—a review focuses on food safety and environmental sustainability in Bangladesh," *Environmental Science and Pollution Research,* vol. 29, no. 3, pp. 3230-3245, 2022.

[6] IQAir., "Dhaka Air Quality Index (AQI)'. Air Quality Index (AQI) and PM2.5 Air Pollution in Dhaka.," 1 February 2023 2023. [Online]. Available: https://www.iqair.com/bangladesh/dhaka.

[7] M. R. S. Pavel, M. Tarannum, S. U. Zaman, F. Jeba, and A. Salam, "Health Risk Assessment of High-Level Particulate Matter Exposure in Different Environments in Mega City Dhaka, Bangladesh," *Bangl. J. Sci. Res.,* vol. 2, pp. 78-87, 2020.

[8] M. Balali-Mood, K. Naseri, Z. Tahergorabi, M. R. Khazdair, and M. Sadeghi, "Toxic Mechanisms of Five Heavy Metals: Mercury, Lead, Chromium, Cadmium, and Arsenic," (in eng), *Front Pharmacol,* vol. 12, p. 643972, 2021, doi: 10.3389/fphar.2021.643972.

[9] P. J. Landrigan *et al.*, "Pollution and children's health," (in eng), *Sci Total Environ,* vol. 650, no. Pt 2, pp. 2389-2394, Feb 10 2019, doi: 10.1016/j.scitotenv.2018.09.375.

For peer review only - http://bmjopen.bmj.com/site/about/guidelines.xhtml

[10] M. L. Ackland *et al.*, "Metals in the Environment as Risk Factors for Infectious Diseases: Gaps and Opportunities," in *Trace Metals and Infectious Diseases*, J. O. Nriagu and E. P. Skaar Eds. Cambridge (MA): MIT Press, 2015.

[11] J. L. Davis and W. Checkley, "Characterization of Air Pollution Exposures as Risk Factors for Tuberculosis Infection," (in eng), *Am J Respir Crit Care Med,* vol. 204, no. 10, pp. 1130-1131, Nov 15 2021, doi: 10.1164/rccm.202107-1795ED.

[12] C. A. Dimala and B. M. Kadia, "A systematic review and meta-analysis on the association between ambient air pollution and pulmonary tuberculosis," (in eng), *Sci Rep,* vol. 12, no. 1, p. 11282, Jul 4 2022, doi: 10.1038/s41598-022-15443-9.

[13] United Nations Statistical Division, "3.3.2 Tuberculosis Incidence’," Global SDG Indicator Platform, 31 October 2018. 2018. [Online]. Available: https://sdg.tracking-progress.org/indicator/3-3-2-tuberculosis-incidence/.

[14] A. U. Anka *et al.*, "Potential mechanisms of some selected heavy metals in the induction of inflammation and autoimmunity," *European Journal of Inflammation,* vol. 20, p. 1721727X221122719, 2022/09/01 2022, doi: 10.1177/1721727X221122719.

[15] J. A. L. Bird, Yue%A Rahman, Hossain Zillur%A Rama, Martin%A Venables, Anthony J., *Toward Great Dhaka: A New Urban Development Paradigm Eastward* (Toward Great Dhaka: A New Urban Development Paradigm Eastward).

[16] NTP, "National Guideline and Operational Manual for Tuberculosis," Dhaka, Bangladesh., 2021.

[17] K. J. Rothman, S. Greenland, and T. L. Lash, *Modern epidemiology*. Wolters Kluwer Health/Lippincott Williams & Wilkins Philadelphia, 2008.

[18] J. H. Lubin, M. H. Gail, and A. G. Ershow, "Sample size and power for case-control studies when exposures are continuous," (in eng), *Stat Med,* vol. 7, no. 3, pp. 363-76, Mar 1988, doi: 10.1002/sim.4780070302.

[19] S. Shah-Kulkarni *et al.*, "Prenatal exposure to mixtures of heavy metals and neurodevelopment in infants at 6 months," *Environmental research,* vol. 182, p. 109122, 2020.

Figure 1: Proposed causal pathway, adapted from existing literature [4, 8-11].

For peer review only

For peer review only - http://bmjopen.bmj.com/site/about/guidelines.xhtml

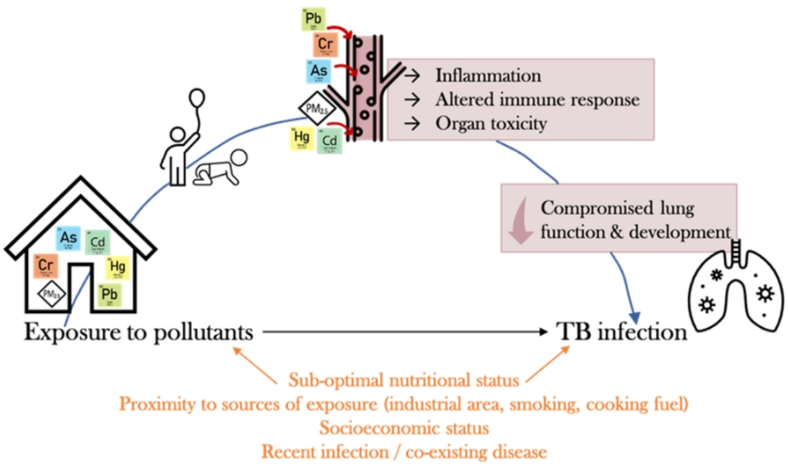

Figure 1: Proposed causal pathway, adapted from existing literature [4, 8-11].

855x481mm (38 x 38 DPI)
